# Examining Sound, Light, and Vibrations as Tools to Manage Microbes and Support Holobionts, Ecosystems, and Technologies

**DOI:** 10.3390/microorganisms12050905

**Published:** 2024-04-30

**Authors:** Rodney R. Dietert, Janice M. Dietert

**Affiliations:** 1 Department of Microbiology and Immunology, Cornell University, Ithaca, NY 14853, USA; 2 Performance Plus Consulting, Hereford, AZ 85615, USA; maninmirror90@gmail.com

**Keywords:** sound, acoustics, cymatics, light, quantum bacterial antennae, microbe-based technologies, energy transitions, Internet of Microbes, holobiont healing, safety

## Abstract

The vast array of interconnected microorganisms across Earth’s ecosystems and within holobionts has been called the “Internet of Microbes.” Bacteria and archaea are masters of energy and information collection, storage, transformation, and dissemination using both “wired” and wireless (at a distance) functions. Specific tools affecting microbial energy and information functions offer effective strategies for managing microbial populations within, between, and beyond holobionts. This narrative review focuses on microbial management using a subset of physical modifiers of microbes: sound and light (as well as related vibrations). These are examined as follows: (1) as tools for managing microbial populations, (2) as tools to support new technologies, (3) as tools for healing humans and other holobionts, and (4) as potential safety dangers for microbial populations and their holobionts. Given microbial sensitivity to sound, light, and vibrations, it is critical that we assign a higher priority to the effects of these physical factors on microbial populations and microbe-laden holobionts. We conclude that specific sound, light, and/or vibrational conditions are significant therapeutic tools that can help support useful microbial populations and help to address the ongoing challenges of holobiont disease. We also caution that inappropriate sound, light, and/or vibration exposure can represent significant hazards that require greater recognition.

## 1. Introduction

Research into human, animal, and plant holobionts (higher eukaryote–microorganism composites) along with planetary microbial life has demonstrated the importance of being able to support, protect, and manage our Earth’s most predominant lifeform: microorganisms. Humans are fundamentally composed of the host and numerous microbiomes (e.g., gut, skin, and airways). Given the fact that humans and most other holobionts on Earth are majority microbial by several criteria, usefully managing microbes should be a prime directive of virtually every earth-directed scientific discipline and especially every medical/public health provider.

Yet, this is far from the case, especially when it comes to human holobiont health and wellness. Calls for microbiome-first approaches to medicine and public health [1,2], and more inclusion of microbiome considerations in public health initiatives [3], have come during a period when holistic, personalized wellness has been institutionally and increasingly ignored. Other examples involve the lack of protection for microbiomes. Two prominent examples concern the world-wide approval and distribution of the antimicrobial toxicant glyphosate [4], and the continued pervasive inclusion of *Akkermansia*-toxic, food emulsifier obesogens (e.g., polysorbate 80) in most processed foods [5]. The cost of ignoring the microbiome despite evidence of its increasing importance plays out across a lifetime. For example, microbiome seeding, feeding, and balance controls the critical development of the immune and other systems in early life [6,7] and also confers protection against the following: uncontrolled fear with mental health consequences [8], regulation of pain and inflammation [9,10], neurobehavioral disorders [11], age-accumulated oxidative damage reducing telomere length and longevity [12], disrupted circadian rhythms [13], and sleep disorders [14]. In short, persistently ignoring microbes and the human microbiome on a global scale would be expected to degrade and compromise the health and lifespan of humanity.

Because of the need to assign greater priority to the protection of useful microorganisms, we are undertaking a series of reviews considering underappreciated physical factors that readily affect holobiont-connected and other microorganisms. Across Earth’s microorganisms (also called the “Internet of Microbes”), communication among and between microorganisms and their hosts occurs in variety of ways. This was discussed in an early review by Reguera [15]. The communication can be wired (via nanowires) or wireless and includes transmission via sound, light (biophotons), and bioelectron exchanges, as well as electromagnetic and chemical signaling. Examples of these functions in action are evident in the processes of microbial management (e.g., rebiosis), restorative ecology and agriculture, and physiological healing (e.g., the microimmunosome). Importantly, these communication processes are not necessarily independent of each other. For example, Matarèse et al. [16] provided an in-depth discussion of the intrinsic linkage between electromagnetic forces and acoustic vibration.

In the present narrative review we seek to accomplish the following objectives: (1) describe the fundamental properties of microorganisms that shows us a path for improved management of microbes; (2) examine how conscious microbial networks both affect and respond to sound, light, and vibrations; (3) describe the role of sound, light, and vibrational approaches in driving technological improvements; (4) describe how sound, light, and vibrational tools offer great promise for holobiont and ecological healing; and (5) conclude that inappropriate use of or exposure to these physical factors can present a significant hazard for much-needed microorganisms as well as humans and other holobionts.

## 2. Examples of Special Bacterial Functions That Have Holobiont/Systems Implications

### 2.1. Communication at a Distance

Significant evidence exists that microorganisms provide a route through which holobionts can communicate at a distance and make changes based on information that originated at a distance. A prime example of this is among plants, which use soil microorganisms (mycelia) as a communication channel and sentient sentries for early alerts to aphid and other pest attacks [17,18]. Plants separated by distance use this microorganism-enabled communication to arm themselves specifically for the impending insect attack. Additionally, the soil microbiome has been shown to affect plant host defenses in general [19,20]. If plants operate at a distance by using The Internet of Microbes, is this the status quo among other holobionts?

### 2.2. Quantum Bacterial Antenna Networks and Applications

In Dietert and Dietert [21], we discussed the ground-breaking research into complex quantum antennae of specialized bacteria. Specific photosynthesizing bacteria have unique capacities to efficiently collect light energy, rapidly pass the energy through a series of proteins and protein complexes, and effectively transform and transfer this energy over long distances. Wang et al. [22] describe the light-originating energy transfer function of purple bacteria using pairwise protein interactions that result in a remarkably efficient, rapid, and extensive energy distribution system. Kundu et al. [23] found that energy transfer from light-harvesting complexes within *Rhodopseudomonas molischianum* could attain 90% efficiency via the quantum motion of nuclei. The quantum processes involved in antenna-driven energy collection and transfer have been described by a number of researchers [24,25,26].

Engineered antennae systems have also been designed to facilitate such processes as biodegradation. For example, Sezgen et al. [27] have described opportunities for multiscale communications through the engineering of the bacterial antennae systems. Additionally, Chen et al. [28] have discussed using bacterial foraging (BF)–based clustering strategies to improve the lifespan of sensor communication networks. Biohydrogen production also includes bacteria sometimes combined with nanotechnology [29]. Finally, the quantum, purple bacteria, light-harvesting system has inspired researchers to create a related artificial polymeric, supramolecular, and column-based light-harvesting platform that offers not only confined and efficient energy transfer but also full-color tunable emission that is suitable for information encryption applications [30]. This illustrates an example of the specialized-bacterial-function-to-breakthrough-technology development that exists.

## 3. Sound and Light Frequencies in Holobiont Cellular Life

Among the many ways that microbes and particular bacteria and archaea collect information, generate energy, and communicate with each other and holobionts are mechanisms using sound and light frequencies as well as electrical and magnetic fields and signals [15,31]. Of course, within holobionts, these same physical factors can have profound effects on the status of holobiont health. The human body itself generates certain sound signatures [32]. Additionally, externally applied sound frequency vibrations can have significant effects on the whole human, as when applied in vibroacoustic therapy [33,34].

When it comes to light, the human body “glimmers” via the generation of weak photon emissions [35]. Calcerrada and Garcia-Ruiz [36] recently reviewed the literature on ultra-weak photon emissions (UPE) emitted from the human body. The authors stressed that it can be used to gauge the internal status of the individual. Because tumor cells have been found to emit increased UPE compared to non-cancerous human cells of the same type, UPE has been seen as a potentially useful tool in early cancer diagnosis [37]. Also termed ultra-weak bioluminescence, Du et al. [38] described how UPE can be used as an oxidative metabolism indicator and is a useful biomarker for specific areas of health vs. disease (e.g., metabolic, skin, and cancer diseases). The researchers also considered UPE when viewed through the lens of traditional Chinese medicine [38]. Finally, UPE has been advocated as a useful tool to detect mitochondrial function vs. dysfunction [39].

Beyond humans, Prasad et al. [40] showed that alterations in UPE comprise a sensitive signal for injury in plants (*Arabidopsis thaliana*). Processes affecting the levels of UPE in bacteria have also been examined by Laager et al. [41]. One of the more recently developed luminescence technologies is aggregation-induced emissions (AIE). Wang et al. [42] described the ways in which AIE can be used for cell, tissue, and microbe imaging, detection, and monitoring of biomarkers and microbes, as an approach to combat disease.

## 4. The Significance of Vibrations

Vibrations are a fundamental signature of life including that of microbes, as described by Kasas et al. [43]. The activity of microbes and cells has a vibrational signature that is extinguished as the cell dies. Kasas et al. [43] showed that nanomotion detectors can reveal microbial life with great sensitivity, and that the vibration fluctuations are largely extinguished as a microbial cell dies due to chemical or physical agents. The presence and status of even individual microbes can be measured based on vibrations. Raman spectroscopy has been a useful tool to identify phenotypes of environmental microbes based on their specific molecular vibration profile [44]. Since microbes and other cells have their own vibrational signatures, it is not surprising that exposure to externally sourced sound, light, and electromagnetic vibrations produces alterations in microbial populations. Nanovibration has been used as a preventative tool that blocks adhesion and biofilm formation by *Escherichia coli* [45]. This narrative review focuses on the sound and light components of vibrationally induced alterations.

## 5. Sound and Acoustics: Effects on Microbiota and beyond

Because sound is a fundamental component of most biological systems, use of sound to manipulate the status of biological materials is gaining ground as a strategy. In fact, the entire field of the study of sound’s effects on biological and other material is known as cymatics. Attention has also been directed in the application of sound, music, and cymatics toward improving human health. For example, a recent review by Liu et al. [46] focused on sub-megahertz (MHz) acoustical waves and their usefulness for medical diagnostics and therapeutics using micromanipulation-based technologies. Sound frequencies are proving useful in both the detection [47] and treatment of human disease [33,48,49]. Examples of diseases and conditions where sound frequency therapy appears promising are the treatment of Parkinson’s disease [50] and other neurological conditions [51] as well as the promotion of wound healing [52].

Sound frequencies are known to play a key factor in communication among microbes, interkingdom communication, and regulation of individual microbes and microbial communities [18,53,54]. One of the early studies on the use of sound by bacteria for communication and on the impact of different sound frequencies on bacterial responses was conducted by Matsuhashi et al. [55]. Such early studies have led to the realization that sound is a tool that can specifically manage microbial populations both increasing the effectiveness of microbes for industrial purposes and promoting improved health of both holobionts (including humans) and even large ecological communities. Znidersic and Watson [56] recently described how sound applications could be used to restore damaged landscapes through the return of interkingdom populations including microorganisms.

The fundamental connection between sound and microbes means that much greater attention is required concerning sound and microorganisms. Protection against deleterious exposure to certain sound frequencies is critical to protect microbes involved in human, animal, and plant health and those supporting ecological media (e.g., soil) and landscapes. Acoustic frequency and strength matters, as per the microbial outcomes. For example, Keramati et al. [57] illustrated in their review that ultrasound (greater than 20 kHz) exposure can produce destruction or alteration of many bacteria while increasing the growth of yeast, and infrasound (frequency below 20 Hz) can likewise decrease certain bacteria’s growth but increase the growth of other microbes. In turn, sound frequencies can be used to optimize a variety of applications including the following: rebiosis/reversing microbial dysbiosis-promoted disease as well as aspects of everyday life (e.g., fermented food and beverage production, enhanced soil for crops/gardening, microbe-driven pollution cleanup, fuel cell efficiencies, and other bioelectric generation applications). Finally, it is important to recognize that sound and light may be more connected than generally assumed [58]. For example, Kassewitz et al. [59] demonstrated that when dolphins focused elocution sounds on specific objects, the reflected sound was captured as images on a CymaScope and displayed as both 2-D and 3-D visuals of the exact same objects. Their sounds have embedded within them the visual image of their focus. Hence, there is a cymatics connection between an auditory sound and a specific visual object that embodies the specific sound.

Table 1 illustrates examples of both review articles and research studies on auditory sound affecting microbial populations [15,16,56,57,60,61,62,63,64,65,66,67,68,69,70,71,72].

There are two extremes of sound frequencies that can play significant roles in affecting microbial populations. These are the sounds above the general human hearing range, termed ultrasound, and the sound frequencies below human hearing, termed infrasound. Ultrasound frequencies (greater than 20 kilohertz, kHz) have been used extensively for decades in medical imaging [73,74] and food preservation applications [75]. Infrasound frequencies (below 20 Hz) extend to below the normal human hearing range [76] but are in the range used by several large mammals (e.g., baleen whales and elephants) and birds [77,78,79]. The issue of safety is always a concern. It should be noted that different human organs and tissues are reported to possess specific vibrational frequencies normally falling in the infrasonic range [34,80]. This may explain why sound and vibration therapies are a logical progression for correcting dysfunctions [34]. Microbial beats (sound vibrations from the human microbiome) have been incorporated with technologies as a strategy of both education and analysis [81]. Vibrational spectroscopy is also proving to be useful for microbial analysis in disease vs. healthy comparisons [82].

Table 2 provides examples of ultra- and infrasounds and microbe alterations [83,84,85,86,87,88,89].

## 6. Light- and Radiation-Frequency Modulation of Microbiota

The study of light-frequency modulation of microbes and other living organisms falls under the general term photobiomodulation (PBM) [90]. As described by Santos et al. [91], photobiomodulation traces back at least to Finsen who won the Nobel prize in Medicine and Physiology for his light-based treatment of both cutaneous tuberculosis and smallpox [92,93]. The term photobiomodulation has become associated with therapy using nonionizing light sources (e.g., LED, lasers, and broadband light) in the visible and infrared spectrums [91,94]. The therapeutic frequencies encompass a range of approximately 600–1200 nm with different frequencies having different skin penetration capacities [91]. Photobiomodulation therapy has been shown to have applications ranging from the treatment of inflammatory and metabolic diseases [95] to dermatological diseases [96], neurological conditions [97], and oral diseases [98]. Anytime misregulated inflammation is being addressed with therapies, it is important to look at the microimmunosome as an initiation point of inflammatory regulation [12,99]. Microimmunosome status is also connected to global intersystem interactions such as those that control circadian rhythms and sleep [12]. Hence, awareness of environmental light exposures and their optimization (e.g., minimizing light-driven circadian disruptions), as well as specific light therapies, are complimentary for overall wellbeing and health.

As with most of the physical–chemical factors discussed in this review, the impact of light on microbes depends upon the nature and contact of the specific microbial population/community and the frequency, intensity, and duration of the given light exposure. In this regard, we provide examples of the range of effects within a narrative review rather than an exhaustive consideration of the massive range of microbes and the full range of different exposures to light.

Different spectra, intensities, and durations of radiation/light exposures can have different effects on microorganisms. Antimicrobial light and radiation exposure represent a major approach to provide food safety and various anticontamination strategies. For example, Shahi et al. [100] provided a comprehensive review of the capacities of radiation and light emission to inactivate viruses and microorganisms in food processing and other routes of pathogenic transition. For nonionizing radiation, microwave, ultraviolet, infrared, laser light, and radiofrequency were considered. Ultraviolet light exposure has long been an approach for microorganism inactivation. Masjoudi et al. [101] reviewed the comparative sensitivity of bacteria, protozoa, viruses, and additional microorganisms to UV-light exposures drawing upon 250 different studies of UV antimicrobial experiments. Li et al. [102] used multibeam excitation and multiwavelength irradiation to inactivate pathogenic microorganisms in water. The emission treatment was found to produce high-efficiency DNA damage and reduced repair while causing membrane damage via reactive oxygen species generation.

In contrast to broad band UV strategies for microbe inactivation, a recent clinical pilot study on human female volunteers conducted by Bosman et al. [103] demonstrated that exposure of skin to narrow-band ultraviolet light shifted the gut microbiome, significantly increasing both alpha diversity (diversity within a sample) and beta diversity (diversity between samples) in the nonvitamin D-supplementing group, enriching populations of *Lachnospiracheae*, *Rikenellaceae*, *Desulfobacteraceae*, *Clostridiales vadin* BB60 group, *Clostridia* Family *XIII*, *Coriobacteriaceae*, *Marinifilaceae*, and *Ruminococcus.* A significant increase in serum 25(OH)D concentrations was also found in the nonsupplementing group, and this increase was correlated with the relative abundance of *Lachnospiracea.* Increased gut microbiome abundance of *Lachnospiraceae* was also observed by Ghaly et al. [104] following skin exposure in mice to narrow-band (311 nm) ultraviolet light. Narrow-band ultraviolet light phototherapy has also been reported to be effective in skin microbiome management of inflammatory allergic dermatitis, as reviewed in Dewi et al. [105].

In a recent study, phototherapy treatment (blue LED light with a peak wavelength of 425–475 nm) of jaundiced infants was found to significantly change the gut microbiota profiles (fecal samples) and secondary bile acid profiles. Infants in treatment for jaundice who received antibiotics differed in their gut microbiota profiles from those receiving light therapy without antibiotics [106]. Additionally, Santos et al. [91] provided a recent review of photobiomodulation therapy as it applies to the human microbiome with an emphasis on red or near-infrared light treatments and the vaginal microbiome.

Light can affect signaling, metabolic activities, and intra-kingdom vs. inter-kingdom communications involving microbes. For example, Xi et al. [107] found that soil-microbe feedback loops guide plant (tree) seedlings in their overall competition depending upon light intensity, the specific mix of soil microbes, and the nature of the plant community (e.g., competitive or noncompetitive trees). Results from the study can help to guide strategies involving light and soil microbes in the restoration of ecologically damaged areas.

Table 3 illustrates examples of the effects of light on microorganisms [91,94,95,103,108,109,110,111,112,113,114,115,116,117,118,119,120,121,122,123,124,125].

The studies and reviews in Table 3 illustrate several key points: (1) light (duration and type) dramatically impacts circadian rhythm, and this is significantly linked to microbiome status and risk of disease. The microbiome, circadian clock, and aging linkage was previously stressed by us [12]. (2) The type of light is critical, and LED white light is not beneficial for the human microbiome or for health. (3) Light pollution can alter the microbiome and increase the risk of inflammatory-driven diseases. (4) Both infrared and ultraviolet light can be therapeutic for microbiome dysbiosis and certain disease conditions. Light exposure of the skin effects not only the skin microbiome but also the gut microbiome. (5) Light exposure impacts both the microimmunosome and the gut–brain axis. (6) Light conditions and treatments apply to human microbiome and human health as well as to the parallel in agriculture (production animals, plants, and soil) and environmental ecosystems. Light-based therapies represent a powerful tool for microbe management as well as for disease therapy. Attention to light conditions is critical for safety to avoid human, agricultural, companion animal, and/or ecological damage.

## 7. Conclusions

Fundamental quantum properties of microbes, as demonstrated most widely in bacteria, provide a ready path to microbial management not only within holobionts but also across ecological and planetary scales. This is illustrated in our present narrative review of two key microbial properties: sound and light, and the capacity of microbial populations to respond to externally applied sound and light frequencies and associated vibrations. Because microbial populations are key to human and other holobiont health and wellbeing, and because they are also integral to ecological and biogeochemical status of the planet, useful application of sound and light approaches are likely to be of greater importance in the near future. Knowledge and appropriate use of these tools is critical to ensure that holistic holobiont healing and well-being is achieved, and that holobionts as well as needed ecological microbes are not damaged from hazardous, inappropriate exposures to the same physical fields. The present review also emphasizes the interconnectedness of Earth’s microbial populations via both wired and wireless information flow via the Internet of Microbes. As a result, both local and at-a-distance effects of physical field changes should be expected and anticipated.

Consideration of sound and light as well as electric and magnetic approaches for human and other holobiont health takes on an added importance given the underperformance of pharma-based Western medicine relative to chronic disease cures [1]. In a series of recent publications, we argued that since the mid-20th century, pharma-driven medicine and public health have not only failed to reduce the prevalence of chronic diseases but have also overseen the growth of polypharmacy and human microbiome and microimmunosome degradation [2,8,126,127]. Hence, it is a useful time to seek alternatives [128]. For this reason, it has become more important than ever to expand the range of microbiome-supportive health and wellness strategies that allow us to manage microbes not only in the human holobionts but across the network of microbial reservoirs on the planet.

This narrative review builds upon a prior review dealing with ancient and alternative healing modalities that have been shown to produce modifications in holobiont microbiomes and/or microbial populations. The significance of the present narrative review is the focus on two functions used by microorganisms to interact with the environment and each other: sound and light. These two field-based approaches to microbe management are also important in technologies ranging from environmental remediation to sustainable energy and future agriculture. One can expect that, just as these tools are having a positive impact on sustainable living, their expanded application to human holobiont health and wellness will be key to microbiome-inclusive medicine.

Finally, it seems clear that future research must look beyond just the microbes bounded by the human body and consider the ways in which inter-holobiont and holobiont–ecological microorganism connections are affected by physical changes in sound, light, vibrations, and electric and magnetic fields. The Internet of Microbes is real [129] and microorganism research shows us that we are truly not separated from Earth’s microbes.

## Figures and Tables

**Table 1:** Examples of sound frequencies, cymatics, music, and microbe alterations.

Experimental Study or Review [Citation]	Experimental Approach [Not Applicable (NA) for Reviews]	Major Experimental Findings/ Review Conclusions
Study of the effects of chronic (30-day duration) white noise at different levels vs. background noise on the mouse gut microbiome and other health-related biomarkers [60].	Groups of three-month-old male SAMP8 mice were exposed to different levels of white noise (88 or 98 dB) for 4 h per day for 30 days while control animals received background noise (40 dB) from another chamber. A group of 8-month-old mice was also used as a positive (aging) control. Behavioral testing, tissue analysis, and cecal microbiota were analyzed.	(1) Noise exposure significantly increased the *Firmicutes*/*Bacteroidetes* ratio. (2) At the genus level, noise increased the levels of *Candidatus Jettenia*, *Denitratisoma*, and *SM1A02*. (3) Chronic noise impaired both intestinal and brain endothelial tight junctions and elevated biomarkers for systemic inflammation. (4) Hippocampal amyloid-β was significantly elevated in the noise-exposed groups (vs. controls) and (5) this parameter could be transferred to non-noise exposed recipient mice via fecal microbiota transplantation.
Experimental comparison in South Africa of exposure of wine grape plants to music vs. controls [61].	Wine Grapes, *Vitis vinifera* L. (cultivar “Syrah”), were planted with one group exposed to classical music 24/7 for the entire growing season while the control was out of range of the music. Core leaf microbiomes were compared (via 16S rRNA gene analysis and ITS fragment amplicon libraries).	Music was associated with an altered grapevine phyllosphere microbiota, which exhibited (1) increased abundance of specific bacteria and fungi, and (2), with certain conditions, distinct taxa previously shown to exhibit beneficial characteristics in host resilience and/or wine terroir (taste).
A study on the impact of a variety of different sound frequencies on the growth and intercellular macromolecular characteristics of *E. coli* K-12 [62].	For this in vitro study, within an experimental apparatus, both the sound frequency and intensity level were adjusted with a waveform generator and the amplifying circuit in the soundwave generating unit. Sound frequency varied from 250 to 16,000 Hz and was maintained at a sound intensity level of 80 dB and a sound power level of 55 dB. The level of sound intensity varied from 0 to 100 dB. The sound power level varied from 55 to 63 dB and was maintained at 8 kHz and 80 dB.	Six-hour exposure of *E. coli* K-12 to a frequency of 8 kHz, with an intensity level of 80 dB and a power level of 61 dB produced (1) significantly increased biomass and intracellular macromolecular synthesis and (2) increased length of the *E. coli* K-12 cells.
Experimental study comparing the effects of music vs. white noise on mice [63].	Six-week-old male SPF C57BL/6J mice received a one week adaptation period with three groups used over a 5-week acoustic trial. Groups were as follows: mice with Mozart for two 1.5 h intervals, mice with white noise at the same dB and time intervals, and controls with no extra sound. Extensive growth, behavioral, physiological, and microbiological data were collected.	The music group was significantly elevated in the *Firmicutes*/ *Bacteroidetes* (F/B) ratio while the white noise group had a significantly reduced FB ratio. White noise increased oxidative stress (with reduced antioxidant levels) and decreased immune function (based on cytokine biomarkers).
Study of the effects of different sound frequencies on brewer’s yeast (*Saccharomyces cerevisiae)* growth and volatile metabolite production [64].	*Saccharomyces cerevisiae* strain CLIB382 isolated from a 1950 Irish brewery was used as the microbe. Two sound frequencies were examined (100 Hz and 10 kHZ) plus silence as a control. The intensity was 90 dB with a background of 41 dB. The culture was sampled for growth and metabolites 16 h after inoculation and then every 4 h until completion (approximately 40 h). Twenty-four separate aroma-associated metabolites were quantitated during the fermentation.	Major changes in growth and aromatic metabolites were found with the different sound treatments. The researchers concluded that sound manipulates the fermentation process such that aroma and flavors (e.g., citrus vs. sweet fruit) of beer and other consumer products could be shifted with simple sound treatments.
The study examined the effects of 1000 Hz frequency sound with and without microaeration on poultry litter digestion [65].	The effects of sound (1000 Hz) with and without microaeration on digestion of poultry litter to produce biogas was examined for both efficiency and microbe alteration. Baseline measurements of digestate were taken at six weeks of operation. Beginning at seven weeks of digestion, sound and/or microaeration was introduced daily with further sampling of biogas and microbes conducted at 23 weeks and 42 weeks of operation.	Sound and microaeration significantly increased microbial diversity beyond controls, including an increase in the *Firmicutes*/ *Bacteroidetes* ratio.
Study examining the effects of different sound frequencies on a variety of microbial functions within osmotic microbial fuel cells [66].	Bacteria were stimulated for 5–6 h per day with a sound wave having an intensity of 60–80 dB and a frequency range from 20 to 1000 Hz.	Sound stimulation (1) increased organic matter degradation and power generation from the bacteria-based fuel cell and (2) decreased the osmotic fuel cell start-up time.
Different sound frequencies were tested on growth and secondary metabolite function among halogenic unicellular green microalgae *Dunaliella salina.* The article also provides review information of prior studies across ultrasound, audible sound, and infrasound [57].	Researchers investigated the effects of 100, 200, 500, and 1000 Hz (90 dB intensity) sound on protein biomass and cell division, using both a nitrite-optimized and deficient media. Beta-carotene was quantitated as an important secondary metabolite. Sound was continuous for the last 15 days of an 18-day culture. For control cultures, the sound was below 40 dB.	Most sound frequencies, increased growth with 200 Hz, facilitating maximum growth while minimizing stress damage, and with 1000 Hz decreasing growth.
Study of in situ effects of acoustic music on the motility and swimming ability of *Escherichia coli* [67].	*E. coli* MG1655 was subjected to synthesized music (via musecore) of the Flight of the Bumblebee. Both indirect (on a sold surface) and direct (in a liquid solution) movement was quantitated. Three different music conditions were evaluated: Highfast (329.68–4186 Hz, 250 Beats per minute, BPM), Midfast (55–1760 Hz, 250 BPM), Midslow (55–1760 Hz, 25 BPM) along with a control group.	Motility, average swimming speed, and absolute average velocity significantly increased in the Highfast and Midfast groups. The Midslow group had extensive variability.
A study of the effects of acoustic sound vibrations on *Pseudomonas aeruginosa* [68].	The study used a 100 Hz vibration system to examine vibrational stress and chemicals on *Pseudomonas aeruginosa* strain PAO1 tolerance after a 48 h culture.	Exposure produced increases in the levels of fatty acids and their derivatives, N-acylethanolamines, and quinolones with decreased levels of rhamnolipids. Gene expression was altered with increased expression of *fabY*, *fade*, and *pqsA* genes and a downregulation of the *rhlA* gene.
A study on the effects of Indian classical music on growth, metabolism, and antibiotic susceptibility in microbial cultures [69].	Eight different prokaryotic and eukaryotic microbes were tested using music ranging in frequency from 41 to 645 Hz with a decibel range of 95–110 dB.	For the eight organisms examined (*Xanthomonas* *campestris*, *Chromobacterium* *violaceum*, *Serratia marcescens*, *Staphylococcus aureus*, *Streptococcus * *pyogenes*, *Streptococcus mutans*, *Saccharomyces cerevisiae*, and *Candida* *albicans*), music enhanced growth and antibiotic susceptibility for all organisms except *S. marcescens*.
Study of cell consciousness metabolism in response to different acoustic vibrations among *Escherichia coli* K-12 [70].	The protocol examined the effects of six different time durations (range of 5–30 min.) Two single frequency sounds (500 Hz and 1000 Hz) and Pali chanting natural sounds by monks (range of 200–900 Hz) were used. Culture absorbance rate was used for evaluation of growth/metabolism at different timepoints.	Overall, continuous exposure to the the Pali chant increased growth for the 5–25 min evaluation times.
Review article discussing the significance of bio-acoustic communication among microbes and across kingdom boundaries. It also considers electromagnetic induction of sound [16].	NA	This review is particularly significant in its discussion of sound among microbes as an information communication signal. The authors used the term ”infosome” to discuss initiators of intermicrobe sound communications and the significance of sound communication during stress in the environment. Importantly, the review also considers sound-based communications relative to holobionts.
Review article discussing sound-based communication among bacteria [15].	NA	This review provides a significant consideration of wired and wireless communication among bacteria including examples that suggest that bacteria can enable neighbors to grow in non-permissive conditions by communicating via sound.
Review article covering the effects of anthropomorphic sound and artificial light on microbes. The emphasis is placed on public health considerations [71].	NA	Among 12 papers found on bacteria and anthropomorphic sound, 8 papers were discussed in detail as per protocols and results. Additional studies were reviewed on algae, fungi, and zooplankton.
Systematic review of music and sound influencing specific cell cultures [72].	NA	This is a systematic review of sound and microbial cell culture. An emphasis is placed on examining mechanobiological stimuli and their effects. Vibrations are considered as part of the effect of sound on microbes. Vibrations are given further consideration in a later section of our present narrative review.
Review of acoustical restoration and the potential of using soundscapes to restore microbe-connected, holobiont ecological communities [56].	NA	This review forges important new ground in examining the use of “acoustical lures” to attract microbes as well as multiple higher organisms to acoustically restore ecologically devastated areas. Scalable acoustic restoration is compared vs. seven other restoration approaches.

**Table 2:** Examples of ultra- and infrasound frequencies and microbe alterations.

Experimental Study or Review [Citation(s)]	Experimental Approach [Not Applicable (NA) for Reviews]	Major Experimental Findings/ Review Conclusions
Infrasonic pulsing for foulant removal [83].	The study investigated the use of pulsed infrasound to in situ microbially clean filtration membranes. *Saccharomyces cerevisiae* (yeast) was used for membrane cake formation. Infrasound-induced membrane vibration is thought to be part of the multistep cleaning process. Talc vs. yeast was use in the evaluations.	While optimal frequency and duration of pulsing differed between the two test systems, infrasound pulsing produced a four-fold improvement in the net flux for the experimental talc system. For the yeast system, it resulted in up to three-fold improvement.
Study of infrasound vibrations on *Escherichia coli* K-12 cell proliferation [84,85].	Radioactive labelling [3 H]-thymidine-based cell proliferation assay was used to examine the effects of several different infrasound frequencies (2, 4, 6, 8, and 10 Hz frequency, at 30 dB intensity) with varying exposure durations for wild-type *E. coli* K-12 cells.	These two research publications from the same group showed that infrasound could have stimulatory or inhibitory effects on *E. coli* cell growth depending upon the exposure duration.
Study of focused ultrasound as a key tool to direct engineered bacteria for cancer immunotherapy [86].	Engineered *Escherichia coli* Nissle 1917 (an approved probiotic bacteria that can colonize certain tumors) was equipped with a trial-selected thermal-sensitive repressor element originally derived from other microbes and designed to thermally switch control of immune checkpoint inhibitors in the tumor environment. Focused ultrasound was used to thermally trigger bacterial gene expression. An in vivo trial was performed against tumors transplanted into female BALB/cJ mice aged 8–12 weeks old.	Following successful in vitro trials of the engineered bacterium, an in vivo trial using tumor- transplanted mice and an ultrasound trigger produced a significant reduction in tumor volume.
Review on use of ultrasound in microbial-mediated processes such as in fermented foods [87].	NA	This review provides a good basis for an understanding of the importance of ultrasound in stimulating microbial growth and food fermentation when low intensities (vs. microbe damaging higher intensities) are utilized. Ultrasound-induced alterations of metabolic processes are also considered.
The review focuses on the use of ultrasound in dairy products [88].	NA	The review provides useful contrasts of differing intensity/wave amplitude effects on microbial populations among dairy products. It presents a model with high-intensity implosion of microbubbles leading to microbial damage.
Review of sound and ultrasound and their effects on biofilm formation and metabolism among food-related microorganisms [89].	NA	The review covers the bactericidal and antibiofilm effects of ultrasound and also includes sections dealing with growth-promoting sound frequencies for specific microbes. Additionally, it considers the enhanced protection from food-related microbes when ultrasound is combined with other factors (e.g., chelating agents, enzymes, and ozone).

**Table 3:** Examples of light treatment and photobiomodulation (PBM) of microbiota.

Experimental Study or Reviews [Citation]	Experimental Approach [Not Applicable (NA) for Reviews]	Major Experimental Findings/ Review Conclusions
A review of PBM of inflammatory bowel disease (IBD), inflammation, and pain stresses two main paths through which PBM influences the gut microbiome [94].	NA	IBD is one of the microbial dysbiosis-mediated diseases where PBM shows considerable promise.
In a review of PBM and chronic kidney disease, the pathways through which PBM facilitates correction of mitochondrial dysfunction as well as gut microbiome dysbiosis are considered main pathways to health improvement [108].	Gut microbiome status is a key target in Chronic Kidney Disease.	This review is important in establishing the significance of PBM on even end-stage diseases with the gut microbiome being an important route.
In a mouse model, Balb/c mice at 10.5 weeks of age were treated with sham, single, and multiple (3× per week) laser treatments using lasers at 660 nm (red) or 808 nm (infrared) [95].	Abdominal shaved skin was the target and fecal microbiota analysis was compared on fecal pellets collected at 0, 7, and 14 days of treatment. 16S rRNA gene analysis was used.	By day 14 in the trial, infrared (but not red)-light treatment significantly increased a genus of bacteria associated with a healthy microbiome: *Allobaculum*
The effects of narrow-band ultraviolet light skin exposure (3× exposures in one week) on intestinal microbiota were examined in healthy human females who took vitamin D supplementation the entire winter vs. those who did not have prior-winter vitamin D supplementation [103].	Pre- and post-treatment blood and fecal samples (two samples of each from each participant) were obtained for vitamin D and gut microbiota analysis.	Exposure of low vitamin D level participants to narrow-band UVB light produced specific alterations in the gut microbiome. For this group, enrichment was found in *Lachnospiracheae*, *Rikenellaceae*, *Desulfobacteraceae*, *Clostridiales vadin* BB60 group, *Clostridia Family XIII*, *Coriobacteriaceae*, *Marinifilaceae*, and *Ruminococcus*.
In a mouse model, the effects of daily full-spectrum phototherapy were examined in 4-week-old female Balb/c mice (nine hours per day of Full-spectrum therapy for nine weeks) [109].	An ovalbumin (OVA)-induced food allergy model was used. Allergic diarrhea, specific immunoglobulins to OVA, Vitamin D_3_ analysis, and fecal microbiota analysis (16S ribosomal RNA gene amplicon) were used. Fecal microbiota transplantation (FMT) was also used from OVA food-allergic mice to naïve recipients to establish the role of the dysbiotic gut microbiota in the food allergy phenotype. For phototherapy, mice received daily exposure to full-spectrum light for 12 h/day throughout the entire experiment (9 weeks).	Dysbiotic microbiota for food-allergic mice were capable of transferring the OVA allergic phenotype. Phototherapy significantly reduced allergic diarrhea, improved vitamin D_3_ levels, reduced OVA-specific IgE and IgG1 antibody levels, balanced specific cytokines, and significantly elevated the gut microbiome *Firmicutes/Bacteroidetes* ratio.
Researchers presented evidence in a commentary suggesting that both natural skin exposure to sunlight and artificial ultraviolet B (UVB) light have similar effects on the gut microbiome. [110].	The commentary compared data from two different studies.	Both artificial narrow-band UVB exposure and natural sun exposure of skin produced increases in gut microbiome diversity involving the phyla *Proteobacteria*. The authors stressed the importance of natural sunlight in gut microbiome maintenance of diversity (with appropriate phototherapy as an option when optimal sunlight was not available).
In a rat model, the effects of continuous light (24 h) vs. a 12 h light, 12 h dark cycle were compared for changes in microbial communities and physiology as well as for potential health risks [111].	Female Sprague Dawley rats (6 weeks old) were exposed to continuous light or a 12 h light/12 h dark cycle for four weeks (after a one-week acclimation). Hormone profiles, histology, gene expression, and fecal microbiota analysis (using a 16S rRNA gene sequencing protocol) were obtained.	Exposure to constant light (and circadian disruption) was associated with a polycystic ovary syndrome phenotype. This exposure resulted in enriched *Parasutterella* with reduced abundance of *Corynebacterium*, genus *Odoribacter*, and *Acinetobacter*.
In a mouse model, ten-week-old male C57BL/6J mice were exposed to continuous light vs. a 12 h light, 12 h dark cycle to determine the role of melatonin in regulating light-induced microbial dysbiosis [112].	Constant light was found to produce both an obesity phenotype and gut microbiome dysbiosis (elevated *Firmicutes*/*Bacteroidetes* ratio plus shifts in certain genera. The effect of melatonin (50 mg/kg body weight in water) as a protective factor was examined.	Melatonin treatment significantly corrected both the aberrant lipid metabolism and the constant light shifts in gut microbiome distribution.
In a mouse model, the effects of far-infrared (FIR) light were examined on gut microbiota [113].	C57BL/6J mice were exposed for 2 min intervals 3× or 5× during a day to examine the short- and long-term effects on the gut microbiome. Microbiome analysis (ERIC-PCR and 16S RNA amplicon sequencing) was performed. Exposure involved electromagnetic waves of 4–20 mm with 85.61% average FIR emissivity and a photon energy level of 12.4 MeV–1.7 eV applied to the mouse abdomen. A two-hour interval between FIR exposures was used.	FIR treatment resulted in three major effects: (1) a reduction in the prevalence of phylum *Deferribacteres* (composed of several pathogens), (2) a significant increase in the prevalence of beneficial genera (e.g., *Alistipes*, *Barnesiella*, and *Prevotella*), and (3) upregulation of key genes connected to short-chain fatty acid regulation and gut homeostasis.
In a mouse model, light and dark stress (24 h dark vs. 12 h light, 12 h dark, vs. 24 h continuous light) were examined for effects on the gut microbiome and memory function and the plasma metabolome [114].	In C57BL/6J male mice, the three lighting conditions were used over a 12-week period with microbiome analysis at baseline and at 4 weeks intervals and behavioral and plasma metabolic analysis after 12 weeks.	Exposure to continuous light in mice resulted in a significant short-term reduction in memory potential. Gut microbiome increases in *Bacteroidales* and *Rikenellaceae* were seen with exposure to continuous darkness, and *Bacteroidales S24-7* was elevated with exposure to continuous light.
The effects of artificial light at night (ALAN) on the soil microbiome of urban areas were examined [115].	Twenty-nine different soil sampling sites across 10 urban turf parks were used in the vicinity of Ningbo city in China. Artificial light levels were obtained via satellite remote sensing. DNA extraction, Illumina sequencing, and high-throughput PCR were all utilized in the analysis of soil samples.	The 29 sampling sites varied significantly in ALAN intensity. ALAN affected the structures of fungal, bacterial, and protist communities as well as functional profiles and nutrient cycling. ALAN was beneficial for some fungal phytopathogens.
In a study using rats, the effects of infrared light on gut microbiota changes and bone loss were evaluated [116].	Because artificial LED white light does not include infrared light, the researchers investigated the effects of supplementing the LED light with infrared (IR) on both the gut microbiome and on bone-related metabolism. Eight-week-old female Sprague Dawley rats were used with half ovariectomized to simulate a bone loss model. IR supplementation occurred for 30 min each day for the three months of the project.	IR supplementation (1) significantly increased the abundance of *Clostridiaceae* and *Erysipelotrichaceae* bacteria, (2) reduced the abundance of *Saccharibacteria*, and (3) increased bone metabolism which correlated with gut microbiome changes.
In a mouse study, the effects of mid-infrared light on gut microbiota and cognitive decline were examined [117].	Six-moth-old APP/PS1 transgenic mice (compared against controls) were used as a model of Alzheimer’s Disease and cognitive decline to examine the effects of mid-infrared light (MIR) on gut microbiota and learning, memory, and amyloid-β (Aβ) plaque load. Behavioral tests, histopathology, and fecal samples subjected to 16S rRNA gene sequencing and analysis were employed. Beginning at 7.5 months of age after baseline sampling, MIR was administered for one hour each day for 1.5 months before final analyses.	MIR treatment produced (1) increased abundance of *Bacteroidetes* and *Verrucomicrobia*, with (2) decreased *Fimicutes*, and (3) increased bacterial diversity with genus-level effects. MIR treatment also attenuated Aβ plaques and improved memory and learning abilities.
In a study using rats, the effects of light duration as well as natural vs. artificial light on gut microbiota were examined [118].	Male Sprague Dawley rats were exposed to a modified 16/8 h light/dark cycle for 8 weeks. Different groups had different types of light during the 16 h period (artificial light group (AL), natural light group (NL), and mixed light group (MX)). The 16 h period was divided into 13 h of the test lighting followed by 3 h artificial nightlight. Corticosterone and melatonin (the latter used as an indicator of circadian rhythm), gut microbiota composition, weight and food efficiency, and depression-like behavior were evaluated.	For the microbiome comparisons, the genus *Lactobacillus* was more abundant in the MX group compared to the other two groups. For NL, the genus *Lachnospiraceae_NK4A136_group* was more abundant in the MX group. NL and MX groups displayed a lower anxiety level and maintained a higher concentration of melatonin than the AL group.
In rats, the effects of constant light on both gut microbiota and risk of diet-induced progression of steatohepatitis were examined [119].	To examine the effects of light and diet on the microbiome, four groups of male Sprague Dawley rats were evaluated: normal light/dark with standard diet (NL-ND), constant light with standard diet (CL-ND), normal light with a high-fat diet (NL-HFD), and constant light with a high-fat diet (CL-HFD). Metabolic parameters were also evaluated. The experimental period was 16 weeks.	Constant light produced glucose abnormalities and dyslipidemia. The CL-HFD group had significant biomarkers for metabolic syndrome (e.g., elevated inflammation and liver steatohepatitis). Constant light resulted in decreased *Butyricicoccus*, *Clostridium*, and *Turicibacter* levels, decreased butyrate levels, and increased indications of a compromised gut barrier.
In mice, light oscillation effects on gut microbiota were examined [120].	Gut microbiota diurnal composition and functional fluctuations were examined using 5-week-old Balb/c male mice and a two-week treatment of light–dark (L-D) vs. dark–dark (D-D) exposures. 16S amplicon sequencing and PCR amplification on cecal samples was used for microbiota analysis.	A rhythmic oscillation of microbiota was noted in the L-D group but not the D-D group with *Bacteroidia* showing a diurnal fluctuation in the L-D group. For functionality, bacteria motility proteins exhibited day/night changes, but the magnitude of the changes was significantly reduced in the D-D group. The abundance of *Clostridia* was significantly increased in the D-D small intestine.
In laying chickens, the effects of reduced light exposure on gut microbiota were examined [121].	The study examined the role of intermittent photoperiod-induced regulation in the interaction between the host circadian clock and the cecal microbial community. Roman laying hens of 20 weeks of age were distributed in three groups: a normal 16 h light/8 h dark group (control), a group where the 16 h light phase had 4 intermittent photoperiod cycles (Low-I), and a group that had 16 intermittent photoperiod cycles within the 16 h light period (High-I). Cecal sample DNA extraction and 16S rRNA amplicon sequencing analysis were used in the microbiota analysis. Cecal metabolic and serum biomarker analyses were also conducted.	Significant findings were as follows: (1) The intermittent photoperiod affected the composition and structure of the gut microbes, (2) correlations were found between the circadian rhythms of gut microbes and the central and peripheral biological clock, (3) melatonin was the route through which the central biological clock affected the circadian rhythms of gut microbes, and (4) microbial metabolites (such as short-chain fatty acids) were the route through which gut microbes provided feedback to enhance clock gene expression in the hypothalamus, liver, and cecal wall.
Light therapy for canine atopic dermatitis and skin microbiome dysbiosis was examined [122].	The effects of topical 308-nm excimer light were examined relative to canine atopic dermatitis (CAD), the skin microbiome, and skin-barrier health. Treatments were given every week for two months for CASD and nonatopic dogs. A variety of parameters were quantitated.	Light therapy significantly (1) reduced atopic dermatitis, (2) altered composition of the skin microbiome (increased *Actinobacteria* and *Cyanobacteria* phyla), (3) increased microbial diversity, and (4) decreased atopic-associated *Staphylococcus pseudintermedius*. Skin barrier function improved with no adverse effects seen.
The interaction between light exposure and the circadian rhythm of the rhizosphere was examined [123].	The effects of light and the circadian clock on the rhizosphere of rice (*Oryza sativa* L.) were evaluated by growing rice for 60 days and then subjecting it to 72 h of either light–dark (L-D) or dark–dark (D-D) cycles. Soil samples were subjected to RNA extraction and 16S cDNA amplicon sequencing and real-time quantitative PCR.	Microbial activity was significantly higher during daytime light than darkness. No circadian cycling was noted in the D-D samples and these samples had significantly lower activity. In the rhizosphere, the proportion of the taxa with circadian rhythms differed significantly between the L-D and D-D treatment groups. These findings shed light on the regulation of circadian rhythms within the rice rhizosphere.
Review of UV radiation (UVR) effects on skin and skin microbiome in humans [124].	NA	This review stresses the importance of UVR for a healthy skin microbiome as well as the protectant metabolite produced by the skin microbes. It also provides useful information on the skin–gut microbiome axis.
A critical review details the recent evidence for photobiomodulation of the vaginal microbiome including dose, specific spectra of light, and microbiome-driven health effects [91].	NA	The review extends the utility of photobiomodulation beyond the gut microbiome to the vagina, the vaginal microbiome, and vaginal immune defense against pathogens.
Review of phototherapy effects relative to both the human microbiome and disease [125].	NA	The review considers the effects of red light and near-infrared light on both rodents and humans with an emphasis on both the gut microbiome and risk of disease. The authors conclude the following: (1) that this is a promising avenue for disease prevention and treatment and (2) that the application has implications relative to circadian cycle maintenance.

## Data Availability

Data discussed in this review article are available via the cited references.
